# Erosion in the occipital bone caused by the fixation instrument used for posterior atlantoaxial fusion -report of 4 cases-

**DOI:** 10.1186/s40064-015-0845-6

**Published:** 2015-03-21

**Authors:** Fumihiro Arizumi, Tokuhide Moriyama, Toshiya Tachibana, Keishi Maruo, Shinichi Inoue, Takanobu Manabe, Shinichi Yoshiya

**Affiliations:** Department of Orthopedic Surgery, Hyogo College of Medicine, 1-1 Mukogawa-cho, Nishinomiya, Hyogo 663-8501 Japan

**Keywords:** Bone erosion, Occipital bone, Posterior spinal fixation

## Abstract

**Introduction:**

Conventionally, posterior C1-C2 fusion has been performed using a sublaminar wiring technique with a structural bone graft. Subsequent advent of newer fixation devices, such as the C1 lateral mass screw and C1 hook, has achieved more solid fixation with improved surgical outcome; however, in these fixation systems, the protruding end of the metal implant above the level of the atlas may result in a complication due to contact with the surrounding structures.

**Case description:**

Two men and two women whose ages at the time of surgery ranged from 14 to 72 years. A supralaminar hook was used as a fixation device for C1 in two cases, whereas a lateral mass screw (Tan’s method) and an atlas claw hook were employed for one each of the remaining 2 cases. We retrospectively reviewed the clinical features and postoperative course of these patients using the clinical records. Moreover, we measured the protruding height of the instrument above the atlas as well as the Redlund-Johnell (R-J) value on postoperative radiographs. All patients complained of crepitus and/or pain on neck extension. Erosion in the occipital bone was detected on multiplanar reconstruction computed tomography (MPR-CT), whereas plain radiographs failed to reveal the bony change. In those cases, protruding instruments used for C1 fixation contacted the occipital bone resulting in an erosive change at the impingement point. We removed the implant in all four cases after confirmation of solid bony union.

**Discussion and evaluation:**

Two of the four patients complained of occipital crepitus alone without pain. The management options for this condition may be controversial; however, progression of bony erosion may result in perforation of the occipital bone. This may possibly be associated with the serious complication of cerebrospinal fluid leakage. Considering this potential sequela, we removed the implants from all our reported cases after confirmation of solid bony union.

**Conclusions:**

We treated four cases that developed erosion in the occipital bone after posterior spinal instrumentation was performed for upper cervical lesions including C1. MPR-CT was useful in detecting the erosive changes in the occipital bone.

## Introduction

Fixation devices, such as sublaminar wiring and the transarticular screw and hook, have been conventionally used with a structural bone graft as the spinal instrumentation for upper cervical spine fixation including C1. Subsequently, the advent of newer instrumentation systems, including the C1 lateral mass screw (Goel & Leheri 1994; Harms & Melcher 2001; Tan et al. 2003) and C1 claw (Cornefjord et al. 2003) has achieved improved fixation strength and eliminated the need for a structural bone graft. Currently, multiple instrumentation systems for posterior C1-C2 fusion using these newer devices are available and are predominantly used in our practice.

Although the introduction of the newer-generation fixation devices has contributed to reduction of the risk for intraoperative complications associated with invasion into the spinal canal, there still may be surgical complications of novel etiologies. We have treated several cases complicated by erosion in the occipital bone caused by repetitive impingement of the fixation device on the bone following posterior spinal fixation including C1. In this report, we present the clinical features of these patients with discussion of the etiology and management of this complication. To the best of our knowledge, this complication has not previously been reported in the literature.

## Case description

We have been using the atlas claw system, the C1 lateral mass screw, and the supralaminar hook for posterior cervical fixation in our clinical practice since 1997. Thereafter, we have performed 17 procedures in 13 patients using these techniques, including 12 claw systems in six cases, four supralaminar hooks in four cases, and one C1 lateral mass screw in one case. Among those patients, four patients sustained erosion in the occipital bone following surgery.

There were two men and two women whose ages at the time of surgery ranged from 14 to 73 years. The demographics and clinical information for each patient are presented in Table 1. We performed C1-C2 fusion alone in three patients and fixation from the C1 to C4 in one patient with a C2 burst fracture. Regarding the instrumentation system, we used the Olerud systems and OASYS in one and three procedures, respectively. We used a supralaminar hook as the fixation device for C1 in two cases, whereas we used the lateral mass screw (Tan’s method) and atlas claw hook for one each of the remaining two cases.

**Table 1:** **Patients who had occipital bone erosion caused by fixation instrument used for posterior atlanto-axial fusion**

**Case**	**Age/sex**	**Diagnosis**	**Symptom**	**Instrumented level**	**Posterior instrumentation**	**Instrument causing erosion**	**The prominence of rod and hook in occipital area (mm)**	**Postop. Redlund-Johnnel (RJ) value**	**Bony fusion**	**Duration till removal of implant**
**1**	14/man	Nonunion of C2 odontoid fracture (Anderson type II)	Occipitalgia	C1/2	C1: supra laminar hook, infralaminar hook C2: laminar screw	Supra laminar hook	4	40	○	3 years 3 months
**2**	61/man	C2 burst fracture	Occipital crepitus	C1-4	C1: lateral mass screw (Tan technique), infra-laminar hook C2: laminar screw C3,4: LMS	Lateral mass screw	3.3	40	○	1 year 2 months
**3**	62/woman	RA, AAS	Occipital crepitus	C1/2	C1: supralaminar hook C2: laminar screw Magerl screw	Supra laminar hook	3.7	35	○	1 year 2 months
**4**	73/woman	RA, AAS	Occipitalgia	C1/2	C1: Atlas claw hook C2: Magerl screw	Atlas claw hook	3.3	39	○	8 years

In all patients, we identified erosion in the occipital bone on multiplanar reconstruction-computed tomography (MPR-CT) images. Using patient charts, we reviewed the postoperative clinical condition for each patient. The clinical follow-up for each patient ranged from 14 to 96 months.

In the analysis of postoperative radiographs, we evaluated anatomical parameters such as the Redlund-Johnell (R-J) value and level of the proximal end of the instrument protruding above the atlas. In addition, we reviewed clinical sequences including bony union and requirement for implant removal.

The Review Board of our institution approved the study protocol, and we obtained the appropriate written informed consent from all patients. The study was authorized by the local ethical committee and was performed in accordance with the Ethical standards of the 1964 Declaration of Helsinki as revised in 2000.

## Discussion and evaluation

We detected erosion in the occipital bone of three recent cases on MPR-CT an average of 7 months postoperatively (range: 6–8 months). We detected erosion in the occipital bone of one previous case using an atlas claw on MPR-CT 8 years postoperatively. This change in the contour of the occipital bone could not be identified on plain radiographs. All the patients complained of local crepitus on neck extension and occipitalgia was associated with this phenomenon in two patients. Cervical spine fixation was maintained in all cases without any signs indicative of loosening of screws or dislodging of hooks. The average amount of protrusion of the rod and hook above the upper level of the atlas was 3.5 mm (range: 3.3–4.0 mm), and the R-J value measured on the postoperative radiographs averaged 38.5 mm (range: 35–40 mm). We did not note the radiological finding of vertical subluxation in any case.

During the subsequent time course, bony fusion was achieved in all cases. Following the confirmation of solid bony fusion, we removed the implants, and all four patients became asymptomatic in the postoperative period ranging from 14 to 96 months (Table 1).

## Presentation of the representative cases

### Case 1

**Figure 1:**
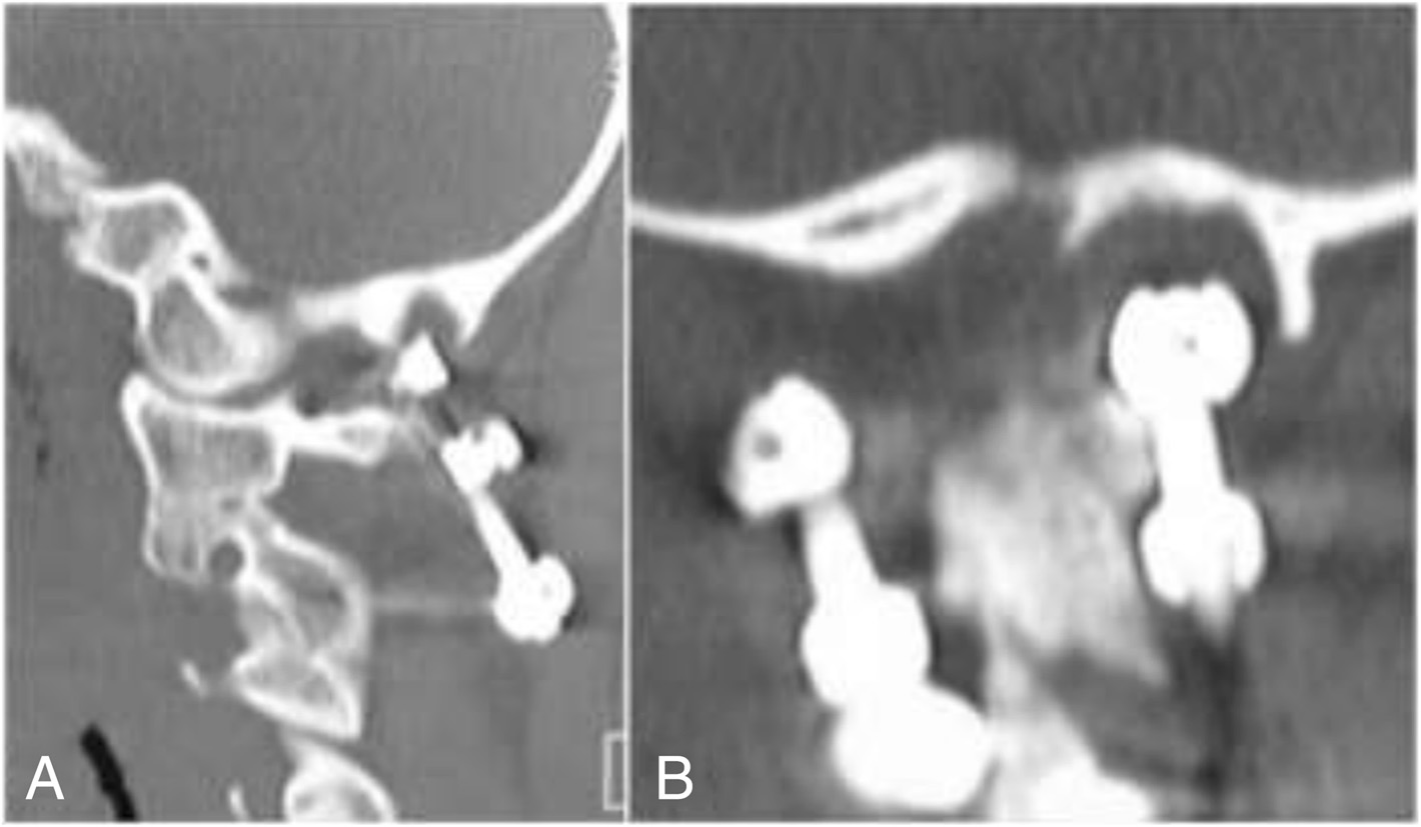
**MPR-CT images of a 14-year-old boy obtained 8 months following C1-C2 fusion using a C1 hook and C2 laminar screw. A**. The sagittal image shows that the supralaminar hook on the left protrudes into the occipital bone. **B**. The coronal image shows that the occipital bone is eroded by the supralaminar hook on the left.

The patient was a 14-year-old boy presenting with occipitalgia and local crepitus at his initial visit. Radiological and CT examinations showed non-union of an odontoid fracture (Anderson type II) that was sustained 4 months before his visit to our institute. He underwent C1-C2 fusion using supra- and infralaminar hooks for C1 and a laminar screw for C2. The postoperative MPR-CT at 8 months showed bony union, whereas erosive changes were identified in the occipital bone corresponding to the location of the tip of the rod of the C1 supralaminar hook (Figure 1).

### Case 2

**Figure 2:**
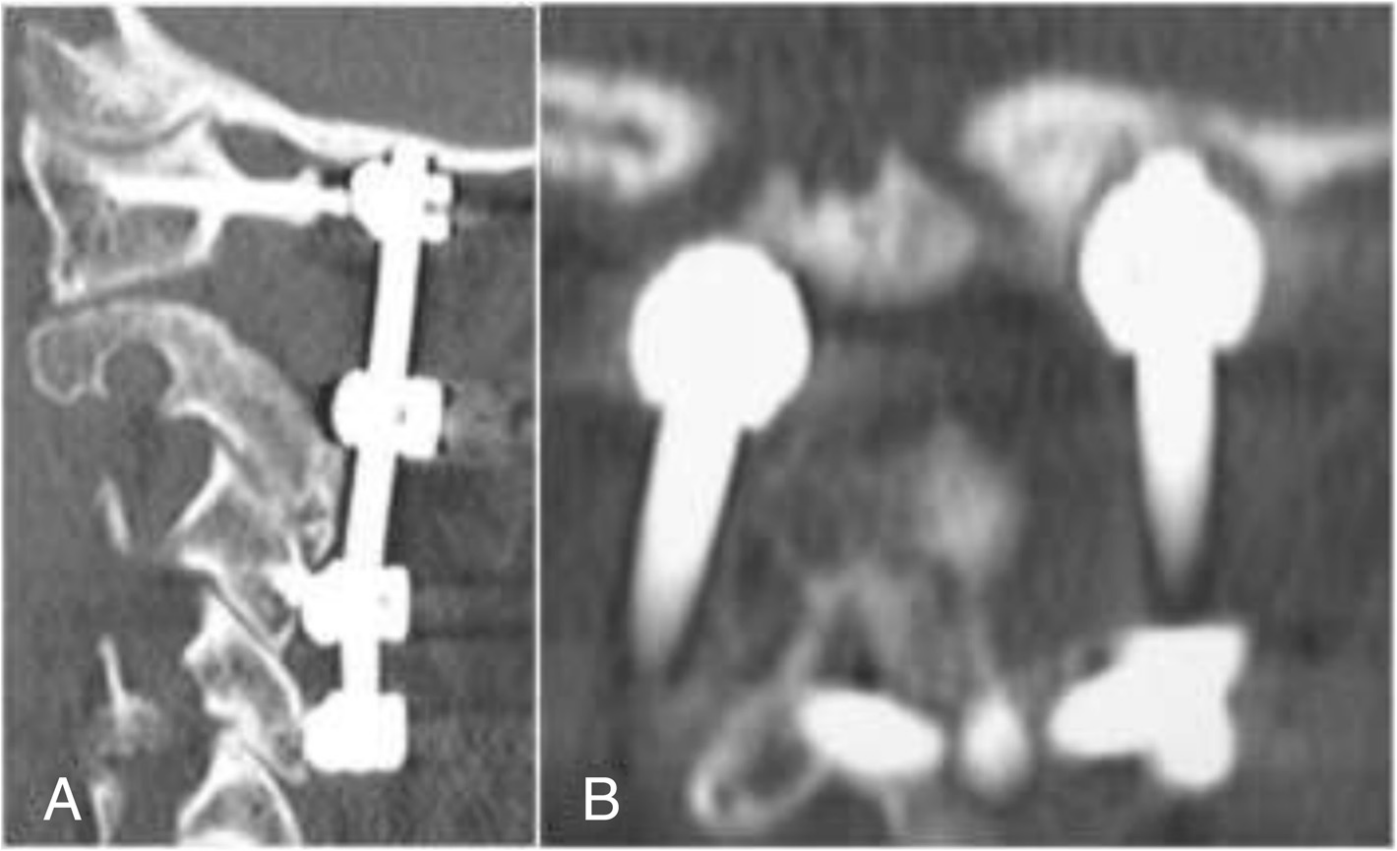
**MPR-CT images of a 61-year-old man obtained at 6 months following C1-C4 fusion. A**. The sagittal image shows that the tip of the rod above the lateral mass screw protrudes into the occipital bone. **B**. The coronal image shows that the occipital bone is eroded by the tip of the rod on the left.

The patient was a 61-year-old man. He had sustained a burst fracture of the axis in a traffic accident and had thereafter suffered from continued pain. We performed posterior spinal fusion from C1 to C4. The devices used for C1 fixation were a lateral mass screw and an infralaminar hook. The MPR-CT at 7 months postoperatively showed erosion in the occipital bone corresponding to the location of the proximal tip of the rod used with the C1 lateral mass screw (Figure 2).

The conventional fixation device used for C1-C2 fusion was a wiring procedure described by Gallie (1937, 1939) McGraw & Rusch (1973) and Brooks & Jenkins (1978). Subsequently, the Halifax clamp (Tucker 1975) hook system and the transarticular screw technique using the Magerl procedure (Magerl & Seeman 1987) have been introduced into practice. These procedures were indicated for various atlantoaxial disorders such as atlantoaxial instability and unstable fracture of the axis. With the intent of further improving fixation properties and eliminating the need for a concomitant structural bone graft, newer methods utilizing fixation of C1 with the lateral mass screw and hook system have been developed and are commonly used in our current clinical practice. Although the advent of these fixation systems has reduced surgery-related morbidity and improved surgical outcome, introduction of novel surgical systems may result in complications that have not been encountered with the conventional procedures. The postoperative complication of erosion in the occipital bone as presented in this paper has not previously been reported in the literature.

The risk of interference with the surrounding tissue using the conventional wiring technique for C1-C2 fusion can be minimal because the fixation construct is not bulky. However, with the use of the devices such as a rod with a C1 lateral mass screw and C1 hook, the proximal end of the instrument may protrude above the level of the atlas. Because the posterior arch of the atlas comes into contact with the occipital bone at maximum cervical extension (Bland 1994) repetitive contact between the occipital bone and metal instrument may result in erosive changes in the bone.

In the reported cases, the instruments responsible for bony erosion were a supralaminar hook in two cases and an atlas claw hook in one case (Cornefjord et al. 2003). When the hook device is used above the level of atlas, the proximal end of the fixation construct may protrude and contact the occipital bone. Thus, the possibility of this reported complication should be considered during the follow-up evaluation. Two procedures have previously been reported in the literature for C1 lateral mass screw fixation: a procedure proposed by Goel & Leheri (1994) and Harms & Melcher (2001) and another described by Tan et al. (2003). The screw entry point in the method of Goel and Harms is at the lateral mass, whereas the screw is inserted via the posterior arch of the atlas in Tan’s method. The insertion point is located at a more proximal level in the latter method, resulting in an increased risk of contact between the instrument and occipital bone. Therefore, surgeons should consider the possibility of postoperative erosion in the occipital bone when using the supralaminar hook and lateral mass screw in Tan’s method. Therefore, the reported cases may be planning error. In reported cases, entry points might have been wrong.

In our cases, the patients complained of crepitus and/or pain in the occipital region. Plain radiographs failed to reveal any erosive change occurring in the occipital bone, whereas MPR-CT clearly identified bony erosion. When patients who undergo posterior C1-C2 fusion with a C1 hook or lateral mass screw complain of symptoms in the corresponding region, the doctor should consider MPR-CT to detect this complication.

Two of our four patients complained of occipital crepitus alone without pain. Management options for this condition may be controversial. However, progression of bony erosion may result in perforation of the occipital bone, which may be associated with the serious complication of cerebrospinal fluid leakage. Considering this potential sequela, we removed the implants from all our cases after confirmation of solid bony union.

## Conclusions

We treated four cases that developed erosion in the occipital bone after posterior spinal instrumentation was performed for upper cervical lesions including C1. We observed that MPR-CT was useful in detecting the erosive change in the occipital bone.
